# Multi-Scale Characterization of High-Temperature Properties and Thermal Storage Stability Performance of Discarded-Mask-Modified Asphalt

**DOI:** 10.3390/ma15217593

**Published:** 2022-10-28

**Authors:** Yuanle Li, Bing Hui, Xinyi Yang, Huimin Wang, Ning Xu, Ponan Feng, Ziye Ma, Hainian Wang

**Affiliations:** 1 School of Highway, Chang’an University, Xi’an 710064, China; 2 College of Mechanical & Electrical Engineering, Henan Agricultural University, Zhengzhou 450002, China

**Keywords:** discarded mask, modified asphalt, high temperature performance, thermal storage stability, molecular dynamics simulation

## Abstract

In the context of the global pandemic of COVID-19, the use and disposal of medical masks have created a series of ethical and environmental issues. The purpose of this paper is to study and evaluate the high temperature properties and thermal storage stability of discarded-mask (DM)-modified asphalt from a multi-scale perspective using molecular dynamics (MD) simulation and experimental methods. A series of tests was conducted to evaluate the physical, rheological, thermal storage stability and microscopic properties of the samples. These tests include softening point, rotational viscosity, dynamic shear rheology (DSR), Fourier transform infrared (FT-IR) spectroscopy and molecular dynamics simulation. The results showed that the DM modifier could improve the softening point, rotational viscosity and rutting factor of the asphalt. After thermal storage, the DM-modified asphalt produced segregation. The difference in the softening point between the top and bottom of the sample increased from 2.2 °C to 17.1 °C when the DM modifier admixture was increased from 1% to 4%. FT-IR test results showed that the main component of the DM modifier was polypropylene, and the DM-modified asphalt was mainly a physical co-blending process. MD simulation results show that the DM modifier can increase the cohesive energy density (CED) and reduce the fractional free volume (FFV) of asphalt and reduce the binding energy between base asphalt and DM modifier. Multi-scale characterization reveals that DM modifiers can improve the high temperature performance and reduce the thermal storage stability of asphalt. It is noteworthy that both macroscopic tests and microscopic simulations show that 1% is an acceptable dosage level.

## 1. Introduction

Since the outbreak of the 2019 coronavirus disease caused by the novel severe acute respiratory syndrome coronavirus 2 (SARS-CoV-2), more infectious mutant strains have emerged [1,2]. Disposable medical masks are gradually becoming an important protective tool for people to protect themselves from viruses, and the massive use and disposal of masks has also brought a series of ethical and environmental issues [3,4]. Masks are mainly made of polypropylene, polyurethane and other polymer materials through a specific production process which makes it difficult for the discarded masks to degrade by natural means [3,5]. In recent years, waste materials such as waste tires, waste straw and waste plastics have become more and more mature in road engineering, which provides a new idea to solve the ethical and environmental problems of discarded masks [6,7,8].

Currently, several studies have emerged on the road performance of discarded masks (DM). Zhao et al. simply crushed the DM and added them to the base asphalt and found that DM could improve the high and low temperature properties of the asphalt [9]. Yalcin et al. found that modified asphalt containing more than 2% DM was better than modified asphalt containing more than 3% SBS in terms of physical properties and rheological performance [10]. Wang et al. melted and crushed DM into fibrous-like form in hot mix asphalt mixtures and found that it can improve the rutting resistance of pavements due to its own structure and stiffness [11]. Goli et al. investigated the effects of different amounts and sizes of DM on the cracking resistance, fatigue resistance and rutting resistance of asphalt mixes, and the results showed that the effect of 12 mm long fibers was superior [12]. It is worth noting that although the performance of discarded-mask-modified asphalt has been studied to some extent, the microscopic modification mechanism is not clear. Moreover, there are fewer studies on the compatibility and thermal storage stability of discarded masks and base asphalt, which limits the further road use of discarded masks.

The molecular dynamics (MD) simulation technique is one of the effective methods to study the microstructure and properties of polymer and asphalt materials. Han et al. studied the effect of the S/B ratio on the interaction between SBS modifier and asphalt using binding energy and diffusion coefficient as indicators, and the results showed that the larger the S/B ratio, the worse the resistance to deformation of the modified asphalt [13]. Guo et al. used solubility parameters and binding energy of rubber-modified asphalt as indicators to study the effect of rubber admixtures and type on the compatibility of modified asphalt [14]. Long et al. analyzed the compatibility of nano-SiO_2_ with asphalt by solubility parameters and Flory-Huggins parameters, and the results showed that nano-SiO_2_ could reduce the volatilization of saturated materials [15]. HU et al. used MD simulation to analyze the interaction mechanism of modified asphalt molecular interfaces in terms of binding energy, mean square displacement, diffusion coefficient and relative concentration distribution [16,17,18,19]. Ren combined MD simulations and experiments to investigate the effect of lignin on the rheological properties of asphalt and found that microscopic thermodynamic indices such as cohesive energy density correlated well with the complex modulus [20].

In conclusion, molecular dynamics simulation is an emerging and effective method to study the microscopic properties and mechanism of polymer-modified asphalt, which plays a verification, complementary and even predictive role for macroscopic tests. The purpose of this paper is to characterize the high-temperature properties and thermal storage stability properties of discarded-mask-modified asphalt at multiple scales. The macroscopic physical properties, rheological properties and thermal storage stability of discarded-mask-modified asphalt were characterized by softening point test, rotational viscosity test and temperature scan test. The microscopic mechanism of discarded-mask-modified asphalt was analyzed by Fourier transform infrared spectroscopy and molecular dynamics simulation. A combination of macroscopic and microscopic approaches was used to investigate and evaluation the performance of DM-modified asphalt and to recommend a suitable DM modifier admixture to promote the road use of discarded masks.

## 2. Materials and Methods

### 2.1. Materials

#### 2.1.1. Base Asphalt

In this study, PUMA 70# base asphalt produced by Starlight Energy (Ningbo, China) Co was used. The main technical properties were tested according to JTG E20-2011 (China), and all the results met the requirements of JTG F40-2004 (China), as shown in Table 1.

#### 2.1.2. Preparation of DM-Modified Asphalt

**Figure 1:**
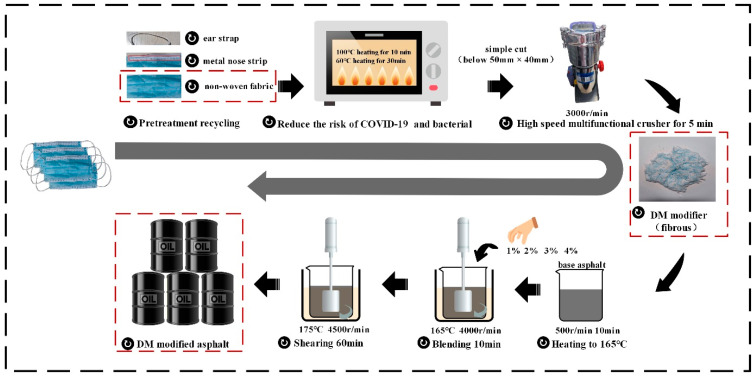
DM-modified asphalt preparation process.

The preparation process of DM modifier and DM-modified asphalt is shown in Figure 1. DM modifier preparation process: firstly, the ear straps and metal nose strips of the discarded masks were removed, and only the non-woven fabric was retained. Then, disinfection and sterilization were performed with reference to previous studies [21,22]. Specifically, the non-woven fabric was placed in an oven and heated at 60 °C for 30 min to reduce the risk of COVID-19 infection and then heated to 100 °C for 10 min to reduce the risk of bacterial infection. Finally, scissors were used to cut the non-woven fabric into pieces (the size is less than 50 mm × 40 mm) and put it into the multi-functional high-speed crusher to crush it for 5 min to get the fibrous DM modifier.

According to previous study [9,10], the DM modifier dosing was selected as 1%, 2%, 3%, 4% of the total weight of the base asphalt. The preparation process of DM-modified asphalt: firstly, the base asphalt was heated to 165 °C and stirred for 10 min at 500 r/min using a high speed shear machine. Then, the DM modifiers with different dosing (1%, 2%, 3%, 4%) were added to the base asphalt with stirred for 10 min at 4000 r/min. Finally, it was heated to 175 °C and sheared at a high speed of 4500 r/min for 60 min. In this way the DM-modified asphalts were obtained. Based on the modifier admixture, the modified asphalt is labeled as DM1, DM2, DM3, DM4.

### 2.2. Characterization Methods

**Figure 2:**
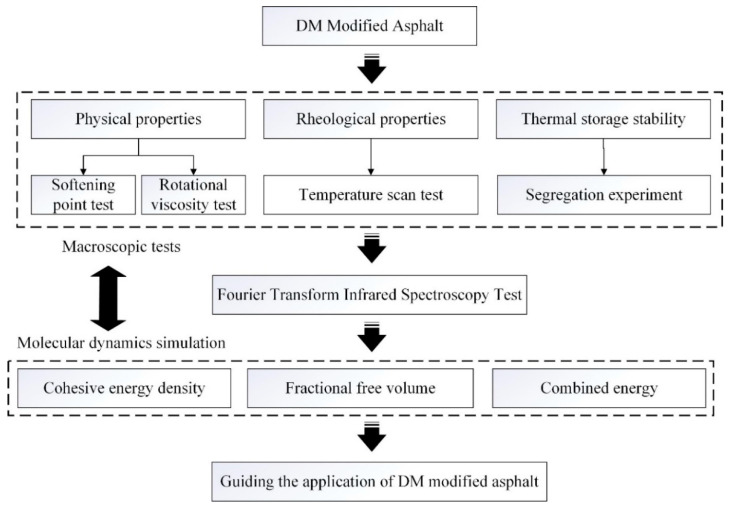
Flowchart of the experimental plan.

In this paper, the high temperature performance and thermal storage stability of DM-modified asphalt are studied and evaluated in terms of physical properties, rheological properties and microscopic mechanism. The research plan is shown in Figure 2, and two samples were taken as a group and the average value was considered as the test result.

#### 2.2.1. Physical Properties

Physical properties can directly reflect the modification effect of DM modifier. In this study, the softening point and rotational viscosity (135 °C, 155 °C, and 175 °C) of the samples were tested according to Chinese standard JTG-E20-2011.

#### 2.2.2. High-Temperature Rheological Properties

Temperature scanning test was carried out using a dynamic shear rheometer (TA Discovery Series HR-3, TA Instruments Waters, Newcastle, DE, USA) according to AASHTO T 315. The sample diameter is 25 mm and the height is 1 mm. The test temperature range is 52 °C to 82 °C at intervals of 6 °C, and the frequency is set to 10 rad/s. The complex modulus, phase angle and rutting factor were obtained for analysis.

#### 2.2.3. Thermal Storage Stability Test

According to Chinese standard JTG-E20-2011 (T 0661-2011) and some previous studies, the thermal storage stability of modified asphalt is evaluated by a segregation test [23,24]. First, the base asphalt, DM1, DM2, DM3 and DM4 were injected into aluminum tubes and sealed, and stored at 163 ± 5 °C for 48 h. Then the samples were left to cool at −5 ± 2 °C for 4 h. Finally, the tubes were cut into three equal parts for softening point testing, rotational viscosity test and temperature scan test. The thermal storage stability of modified asphalt was characterized by softening point difference (Δ*TR&B*), rotational viscosity stability index (VS) and rutting factor stability index (RS), as shown in Equations (1)–(3).

(1) $$\Delta TR\&B=|TR\&B_{t}-TR\&B_{b}|$$

(2) $$VS=(u_{t}-u_{b})/u_{0}$$

(3) $$RS=(G*/sin\delta {)}_{t}/(G*/sin\delta {)}_{b}-1$$

where $u_{t}$, $u_{b}$ are the top and bottom rotational viscosity of the sample after thermal storage and $u_{0}$ is the rotational viscosity of the sample before thermal storage; G* is the complex modulus of the sample; δ is the phase angle of the sample and $(G*/sin\delta {)}_{t}$,$(G*/sin\delta {)}_{b}$ is the rutting factor of the top and bottom parts of the sample after thermal storage.

#### 2.2.4. Fourier Transform Infrared Spectroscopy

In order to determine the main components of discarded masks and whether there is an obvious chemical reaction in the process of modifying asphalt of discarded masks, Fourier transform infrared detection of base asphalt, DM2, DM2_t_ and DM2_b_ was carried out in this study. The detection instrument was Nicolet IS 50/6700 FTIR (Shanghai, China) spectrometer and the wavenumber of 4000 cm^−1^–500 cm^−1^ was taken for analysis.

### 2.3. Molecular Dynamics Simulation

**Figure 3:**
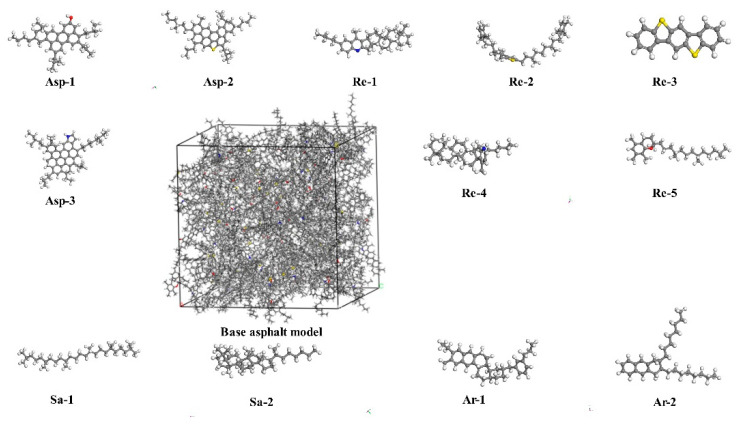
Molecular structure of base asphalt.

In order to reasonably characterize the four components of asphalt, a 12-molecule base asphalt model was proposed by Li et al. [25]. Referring to the previous research results, this paper used Asp-1, Asp-2 and Asp-3 to represent asphaltenes (Asp); Re-1, Re-2, Re-3, Re-4 and Re-5 to represent resin (Re); Sa-1 and Sa-2 to represent saturated (Sa) fractions; and Ar-1 and Ar-2 to represent aromatic (Ar) fractions to construct a base asphalt model, as shown in Figure 3. The relevant parameters of the base asphalt model are shown in Table 2.

The DM-modified asphalt model was constructed based on the actual admixture of DM modifier (1–4%) and the main components of DM modifier determined by FTIR.

1. The asphalt model is constructed using the “Construction” task in the Amorphous cell module to ensure that the asphalt components are randomly distributed in the cells.
2. We optimized the structure in 10,000 steps by using the “Geometry Optimization” task in the Forcite module to minimize the energy of the system.
3. We performed 5 cycles (298–598 K) of annealing optimization for a total of 50 ps through the “Anneal” task in the Forcite module to eliminate local high energy sites in the model.
4. We performed 100 ps of dynamics calculations through the “Dynamics“ task in the Forcite module to fully mix the molecules in the asphalt.

Condensed-phase optimized molecular potentials for atomistic simulation studies (COMPASS) force field can calculate the molecular motion of organic molecular systems more accurately and is often applied to asphalt research [16,26,27]. In this study, the COMPASS force field and one standard atmosphere were used in the model construction and optimization process. The temperature control and pressure control methods were nose and Berendsen, respectively, and the electrostatic energy and van der Waals force were chosen as Ewald- and Atom-based summation methods, respectively.

## 3. Results and Discussions

### 3.1. Physical Properties

**Figure 4:**
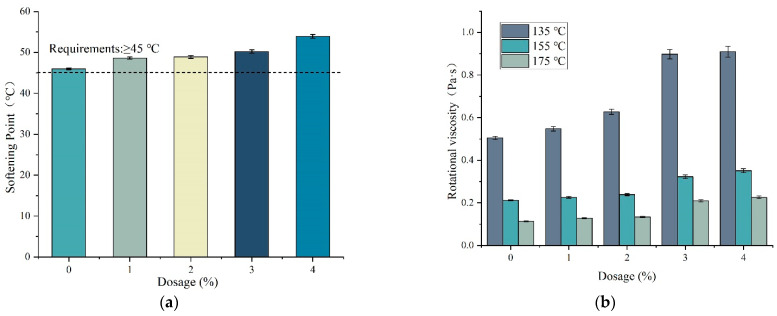
Physical properties of DM-modified asphalt. (**a**) Softening point and (**b**) rotational viscosity (135 °C, 155 °C and 175 °C).

Figure 4 shows the softening points and rotational viscosity of DM-modified asphalt. With the increase of DM modifier, the softening point and rotational viscosity of modified asphalt also increased gradually, which may be due to the swelling of the DM modifier, the absorption of light components in asphalt and the change of the ratio of each component cross-linked with the polymer to form a new architectural system [28]. Compared with the base asphalt, the softening point and rotational viscosity (155 °C) was increased by 5.6%/6.6%, 6.3%/12.74%, 9.1%/52.36% and 17.3%/65.57% for DM-modified admixture from 1% to 4%, respectively. DM-modified asphalt rotational viscosity values are less than 3 Pa·s, which is in line with the requirements of SHRP. The physical experimental results show that the admixture of DM modifier can reduce the temperature sensitivity of asphalt and increase the viscosity of asphalt.

### 3.2. High Temperature Rheological Properties

**Figure 5:**
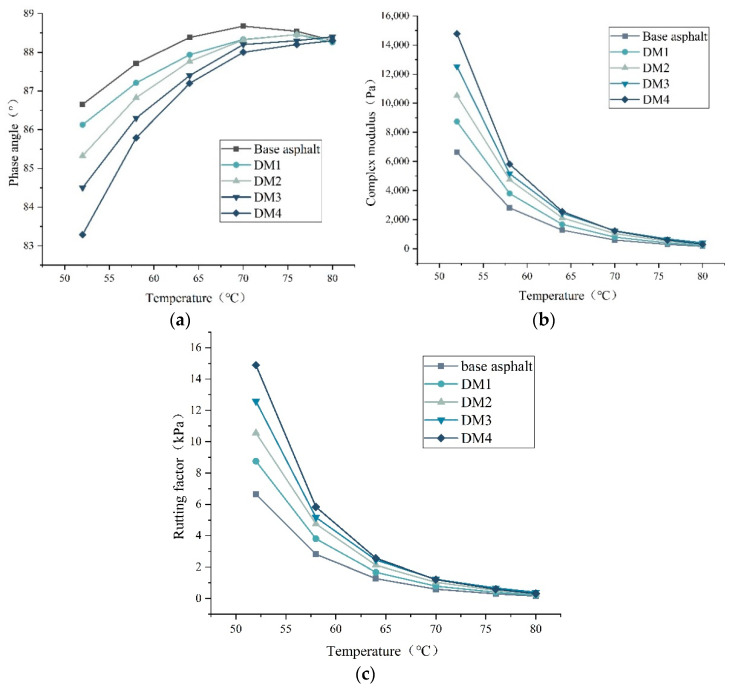
Temperature scanning test results. (**a**) Phase angle; (**b**) complex modulus; (**c**) rutting factor.

Figure 5 show the results of the temperature scans of DM-modified asphalt. The phase angle increases with increasing temperature and the complex modulus has the opposite trend. The phase angle decreases with the increase of modifier dosing, and the trend of complex modulus is opposite. This is consistent with the findings of previous studies [9,10].

The rutting factor of DM-modified asphalt decreases rapidly with the increase of temperature, which is mainly because the increase of temperature causes the viscoelastic asphalt with the proportion of viscous components to increase and the resistance to deformation decrease. The rutting factor of DM-modified asphalt increased with the increase of admixture at the same temperature, indicating that the addition of DM modifier enhanced the high temperature deformation resistance of asphalt. These results are consistent with the previous studies [29,30].

### 3.3. Thermal Storage Stability

**Figure 6:**
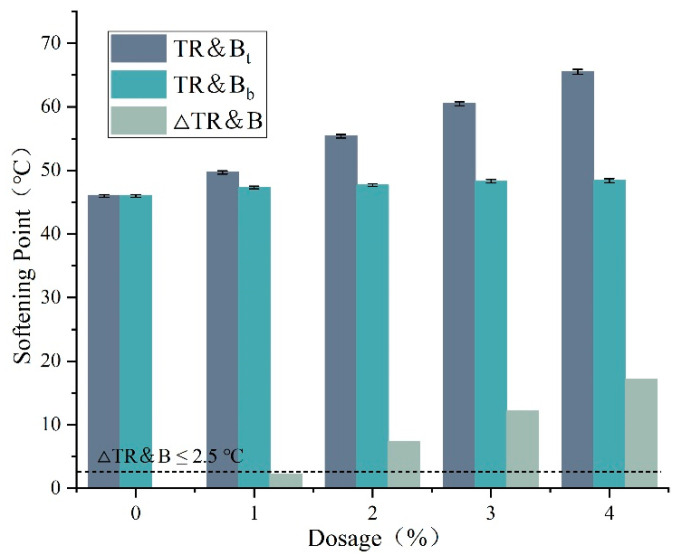
Softening point of DM-modified asphalt after thermal storage.

Figure 6 shows the results of the softening point of DM-modified asphalt after thermal storage. The Δ*TR&B* of DM-modified asphalt increased with the increase of admixture; for example, the Δ*TR&B* of modified asphalt increased from 2.2 °C to 17.1 °C when the admixture of DM modifier increased from 1% to 4%, which indicated that the admixture of DM modifier was unfavorable to the thermal storage stability. The softening point at the top of the samples (*TR&Bt*) was overall larger than the softening point at the bottom (*TR&Bb*) due to polymer aggregation during thermal storage. When the DM modifier dosing is around 1%, the modified asphalt after 48 h hot storage has a Δ*TR&B* less than 2.5 °C, which meets the requirements of Chinese specifications.

**Figure 7:**
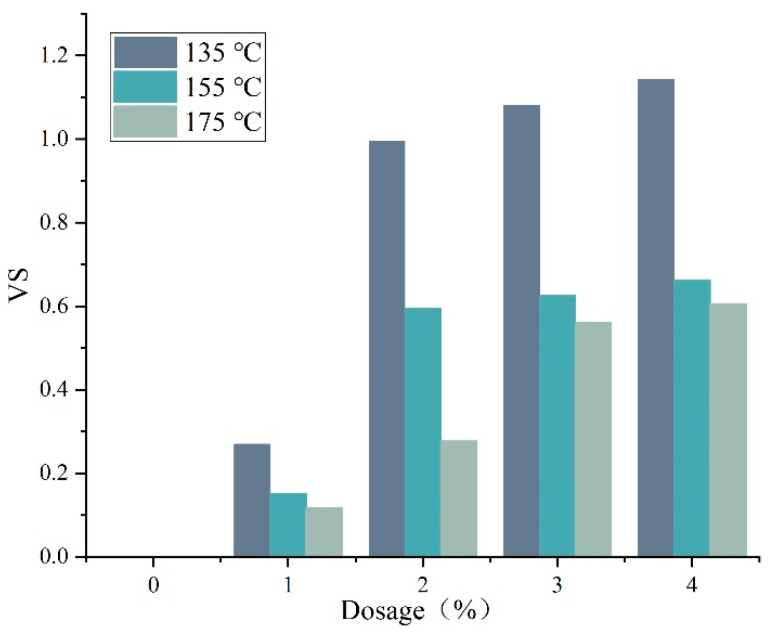
DM-modified asphalt VS index comparison results.

Figure 7 shows the viscosity stability index (VS) of DM-modified asphalt. The closer the VS is to 0, the better is the storage stability of the modified asphalt. VS increases with increasing dosage at the same test temperature. Considering from the perspective of viscosity, the admixture with DM as modifier leads to poor storage stability of modified asphalt. In addition, there is a clear threshold of thermal storage stability of DM-modified asphalt between 1% and 2% of DM modifier admixture. For example, the DM modifier admixture increased from 1% to 2%, and VS increased by 268.5%, 294.0% and 135.9%, respectively.

**Figure 8:**
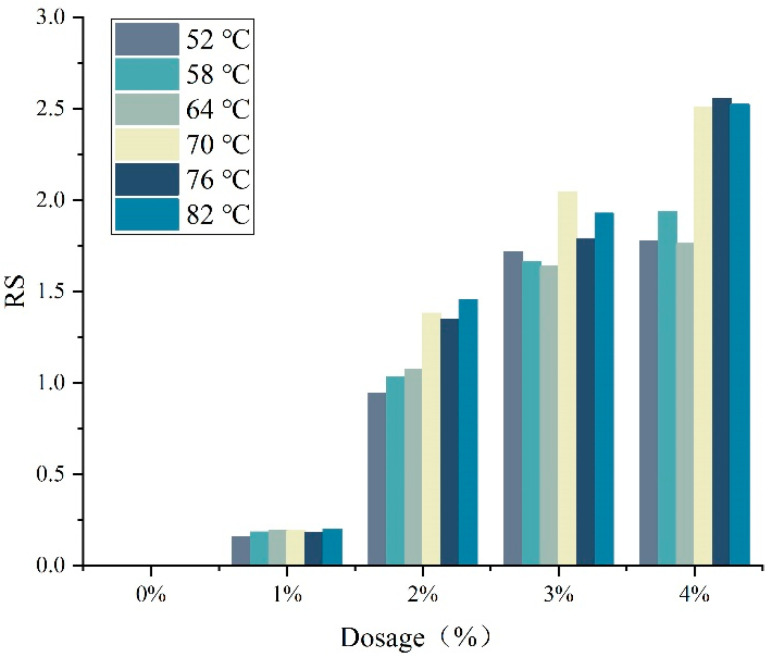
DM-modified asphalt RS index result.

Figure 8 shows the results of RS index of DM-modified asphalt. The larger the RS value, the worse the thermal storage stability of DM-modified asphalt. It can be clearly seen that the RS values of DM-modified asphalt increased with the increase in modifier admixture. This indicates that the rutting factor stability of modified asphalt is worse with the higher dosage of modifier admixture. In addition, at the scanning temperature of 58 °C and as the modifier dose increased from 1% to 4%, the RS values of DM-modified asphalt were 0.184, 1.035, 1.663 and 1.938, respectively. As seen, the softening point difference, VS and RS values obtained from the thermal storage stability test all indicate that there is a critical point for DM-modified asphalt between 1% and 2% modifier admixture.

### 3.4. Fourier Transform Infrared Spectroscopy Analysis

**Figure 9:**
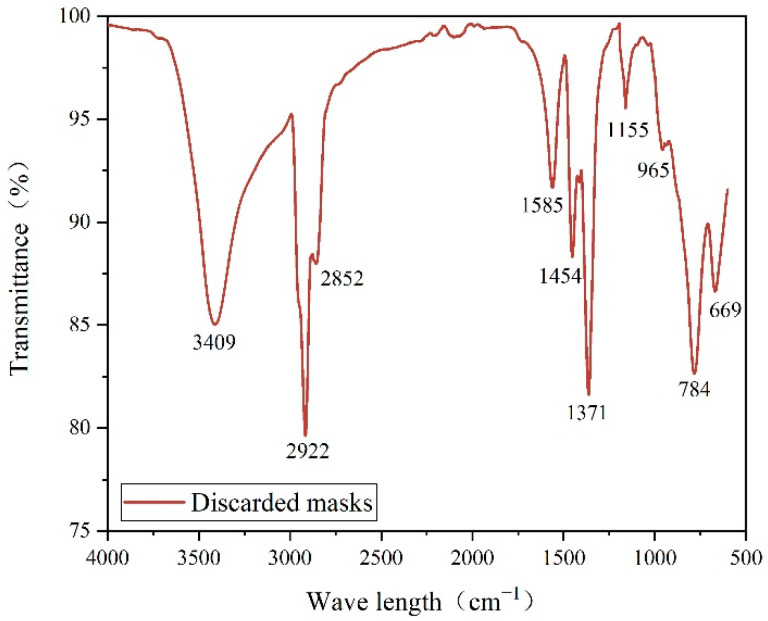
FTIR spectra of discarded medical masks.

Figure 9 shows the FTIR spectrum of the discarded medical masks. The sample showed peaks at 2922 cm^−1^, 2852 cm^−1^, 1454 cm^−1^, 1371 cm^−1^, 1155 cm^−1^, 965 cm^−1^, and the DM was found to be probably composed mainly of polypropylene (PP) material when compared with the infrared spectrum of PP standards [9,31]. In addition, the possible stretching vibration of N-H at 3409 cm^−1^ and the possible bending vibration of N-H at 1558 cm^−1^ suggest that other polymeric materials such as polyester fibers or polyurethane may also be present in the DM [32].

**Figure 10:**
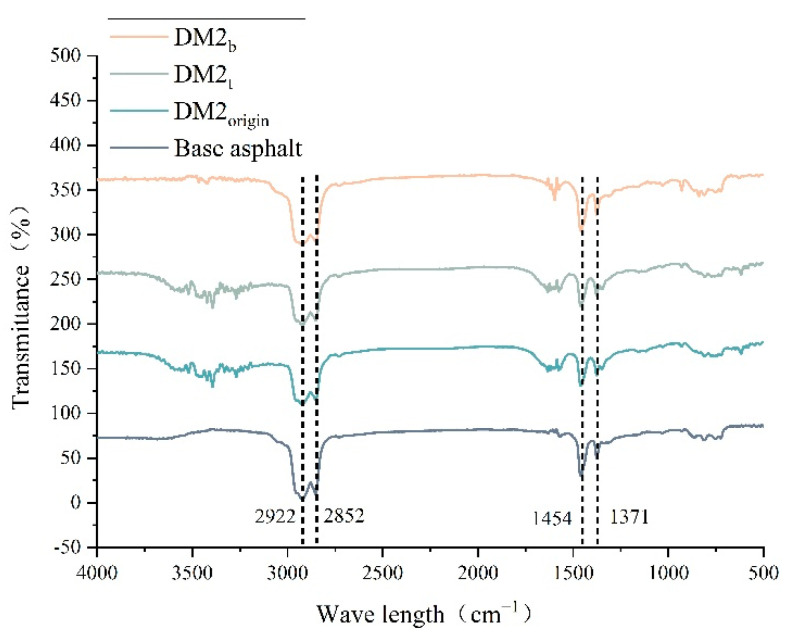
FTIR spectra of DM-modified asphalt.

Figure 10 shows the infrared spectrum of the DM-modified asphalt. DM2_t_ and DM2_b_ are the top and bottom test results of the sample after thermal storage, respectively, and DM2_origin_ is the test result of the sample before thermal storage. After adding DM modifier, strong absorption peaks appear between 2800 and 3000 cm^−1^ and 1300 and 1500 cm^−1^, considering that the absorption peaks at 2922 cm^−1^ and 2852 cm^−1^ are related to the stretching vibration of CH_2_, and the absorption peaks at 1454 cm^−1^ and 1371 cm^−1^ are related to the variable angle vibration of CH_3_. In addition, more pronounced peak fluctuations were observed around 3409 cm^−1^ and 1556 cm^−1^ for the undissociated sample before thermal storage and the top sample after thermal storage, which may be related to the polymer content in the DM modifier. Therefore, from the FTIR analysis, it can be inferred that the use of DM-modified asphalt is mainly a physical co-mingling process.

### 3.5. Molecular Dynamics Simulation

#### 3.5.1. DM-modified Asphalt Model Construction

Based on the experimental results of FTIR and relevant literature [9,10], polypropylene (PP) is the main component of DM modifier, and this paper uses PP to represent DM modifier for molecular dynamics simulation. Due to limited computational resources, it was not possible to introduce the actual degree of polymerization of PP into the MD simulation. The current research results have shown that the solubility parameters remain stable when the polymerization degree in MD simulation reaches a certain critical value [33]. The polypropylene solubility parameter δ is defined as the root of the internal cohesion energy density (CED), as shown in Equation (4).

(4) $$\delta =\sqrt{CED}$$

**Figure 11:**
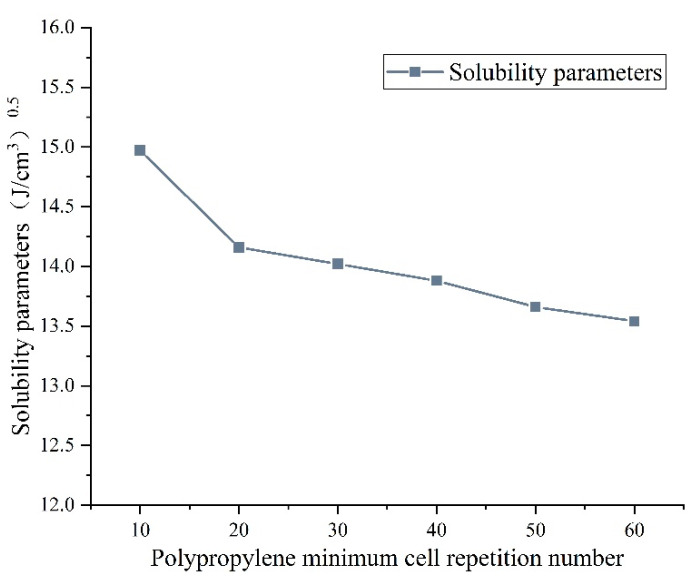
Solubility parameters of polypropylene.

Figure 11 shows the solubility parameters calculated from the simulation of MD of polypropylene with different polymerization degrees. It can be clearly seen that the solubility parameter changes smoothly after the degree of polymerization is greater than 20, which can present PP chains to some extent. In this paper, 20 repeating units are chosen to represent PP chains and its molecular mass is 843.636 g/mol.

**Figure 12:**
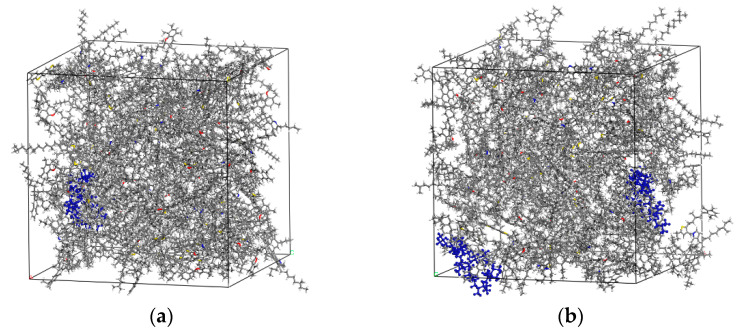

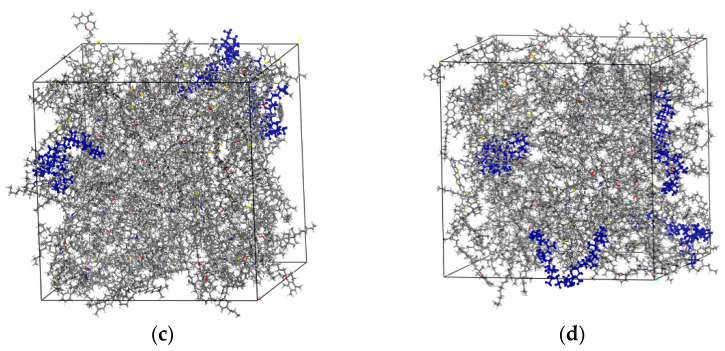
Steady-state DM-modified asphalt model. (**a**) DM1-modified asphalt model; (**b**) DM2-modified asphalt model; (**c**) DM3-modified asphalt model; (**d**) DM4-modified asphalt model. The blue part is the polypropylene chain.

According to the actual admixture of DM modifier in macroscopic tests (1–4%) and the molecular mass of polypropylene, 1, 2, 3 and 4 polypropylene chains were added to the 12-component base asphalt model in this paper. The steady-state modified asphalt model obtained after structural optimization and dynamics calculation is shown in Figure 12.

#### 3.5.2. Model Reasonableness Verification

**Figure 13:**
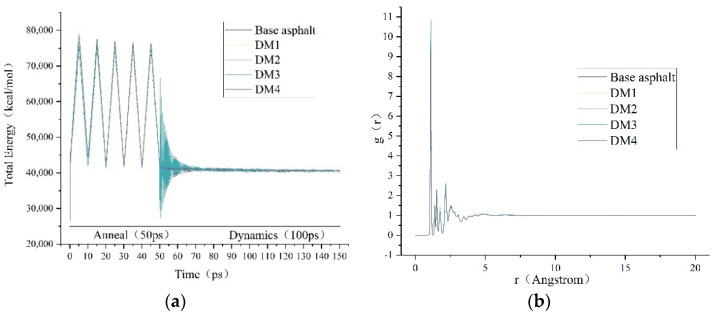
Model reasonableness verification. (**a**) Energy state of the model optimization process and (**b**) radial distribution function (RDF).

Figure 13a shows the energy changes of the base asphalt and DM-modified asphalt models during the optimization process. The total energy of the model system tends to be smooth after annealing optimization and dynamics calculation, indicating that each model constructed in this paper reached a stable state. It is worth noting that the total energy at final equilibrium of the asphalt system increases slightly with the increase of DM modifier admixture. Figure 13b shows the radial distribution function (RDF) of each steady-state model. It can be seen that the g(r) of each steady-state model exhibits sharp oscillation peaks in the short-range range of 0–3 A, and the oscillation amplitude becomes smaller in the range of 3–5 A and is basically stable after the range of 5 A, which indicates that the molecular distribution in the system is irregular and basically conforms to the structural characteristics of the asphalt material [34,35].

Table 3 shows the densities of the asphalt models. The stable density of the base asphalt model reached 0.964 g/cm^3^ at 298 K and 1 standard atmospheric pressure, which is basically consistent with previous studies [36,37]. In addition, the density of the DM-modified asphalt model is slightly smaller than that of the base asphalt, which is consistent with reality. In summary, the asphalt model constructed in this paper has a certain degree of reasonableness.

#### 3.5.3. Cohesive Energy Density

**Figure 14:**
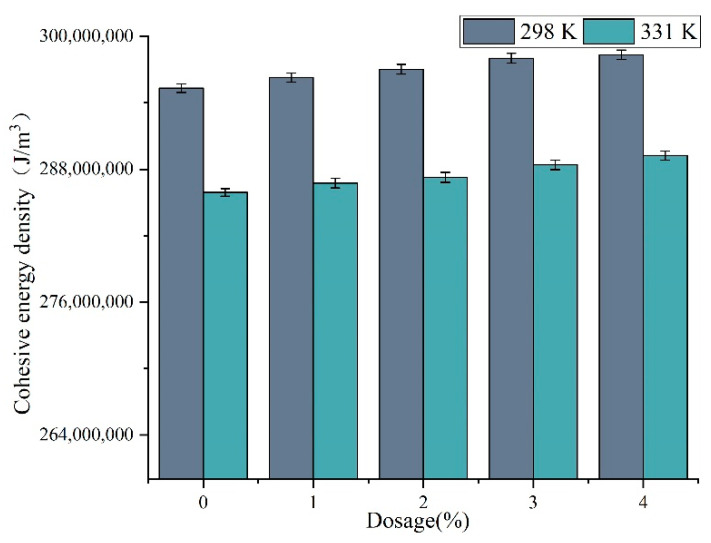
The cohesive energy density of the model.

Figure 14 shows the cohesive energy density for each steady-state model. The cohesion energy density characterizes the magnitude of the cohesion energy of a material per unit volume. The larger the cohesion energy density, the stronger the connection between the molecules inside the asphalt material, which can reflect its resistance to deformation to a certain extent. The results of molecular dynamics simulation showed that the cohesive energy density of DM-modified asphalt increased with the amount of DM modifier at 331 K (around 58 °C). The addition of DM modifier may cause the asphalt to become harder, which is consistent with the conclusion that the rutting factor increases with the amount of DM modifier in the temperature scan experiment.

In addition, the cohesion energy density of each steady-state model at 331 K is generally smaller than 298 K, which may be due to the increase of molecular dynamic energy and the increase of macroscopic volume at higher temperature, resulting in the decrease of molecular cohesion energy density [38]. This phenomenon is consistent with the law that the rutting factor decreases and the deformation resistance decreases when the temperature increases in the temperature scan test.

#### 3.5.4. Fractional Free Volume

**Figure 15:**
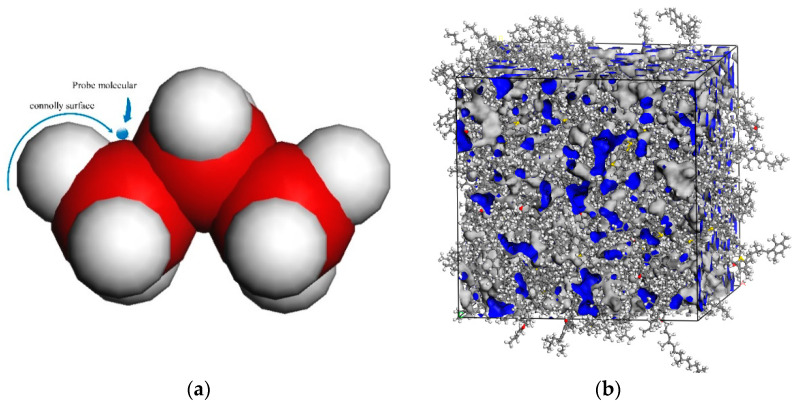
Morphology of the free volume at 331 K. (**a**) Schematic of the free volume calculation method; (**b**) base asphalt.

The free volume is defined as the volume beyond the Connolly surface that the probe can reach, as shown in Figure 15a. The fractional free volume (FFV) is the ratio of the free volume to the total volume of the molecular system, which can be calculated from Equation (5). The smaller the FFV of the asphalt, the less mobile and less permeable are the molecules in the system [39]. Macroscopically, this is reflected in the hardening of the asphalt and its increased resistance to deformation. In this paper, MD simulations are used to calculate the FFV of each steady-state asphalt model at 298 K (around 25 °C) and 331 K (around 58 °C). Figure 15b shows the free volume morphology of the base asphalt. The blue area is the free volume of the system after MD calculation.

(5) $$FFV=V_{f}/(V_{f}+V_{0})\times 100\%$$

where $V_{f}$ is the free volume and $V_{0}$ is the occupied volume.

Table 4 and Table 5 show the results of MD calculations for FFV at 298 K and 331 K for each steady-state asphalt model. With the increase of DM modifier admixture, the FFV of each asphalt system gradually decreases, which indicates that the percentage of free volume in the asphalt system decreases, the asphalt is difficult to compress and the resistance to deformation increases. This is somewhat consistent with the laws exhibited by the macroscopic tests.

#### 3.5.5. Binding Energy

The binding energy is the negative value of the intermolecular interaction energy, which can be calculated by Equation (6) and can reflect the interaction between the components of the mixed system and is often used to predict the compatibility of different materials in the mixed system [14]. The larger the binding energy, the stronger the interaction, indicating higher thermodynamic stability.

(6) $$E_{binding}=-E_{\mathrm{int}er}=-(E_{total}-E_{A}-E_{B})$$

where $E_{total}$ is the total energy of the mixed steady-state system and $E_{A}$ and $E_{B}$ are the total energy of the single-material steady-state system

Table 6 and Table 7 show the binding energy of DM modifier with base asphalt at 298 K and 331 K. It can be found that with the increase of DM modifier dosing, the binding energy of DM modifier and base asphalt shows a decreasing trend, which indicates that the DM modifier dosing has a negative effect on the thermodynamic stability of the mixed system. It is worth noting that the binding energy becomes negative after the dosing of DM modifier is greater than 1%, which indicates that the compatibility between the materials is poor and phase separation easily occurs.

## 4. Conclusions

The purpose of this paper is to investigate and evaluation the effect of DM modifiers on the high temperature performance and thermal storage stability performance of asphalt by a combination of MD simulation and experiment, complementing and verifying each other. Ultimately, a suitable DM modifier admixture for road use is recommended. The main conclusions are as follows:

(1) The DM modifier improves the high temperature deformation resistance of asphalt. The softening point, rotational viscosity, complex modulus and rutting factor of the modified asphalt were increased with the increase of modifier dosing. At the same time, the phase angle of the modified asphalt decreased.
(2) The results of the segregation experiments showed that the addition of DM modifier was detrimental to the thermal storage stability of the asphalt. The DM modifier admixture was increased from 1% to 4%, and the Δ*TR&B* between the top and bottom of the sample increased from 2.2 °C to 17.1 °C, VS (135 °C) from 0.27 to 1.14 and RS (58 °C) from 0.18 to 1.94. It is noteworthy that 1% is an acceptable admixture.
(3) The main raw material in DM modifier is polypropylene, and the modification method is mainly physical blending. The results of FTIR spectra showed that the DM material had absorption peaks at 2922 cm^−1^, 2852 cm^−1^, 1454 cm^−1^, 1371 cm^−1^, 1155 cm^−1^, 965 cm^−1^, which were consistent with the main characteristic peaks of polypropylene. When 2% DM modifier was blended, there was no obvious new characteristic peaks found in the modified asphalt, indicating that the DM-modified asphalt mode is mainly a physical blending.
(4) MD simulation results show that the addition of DM modifier can increase the cohesive energy density (CED) and reduce the fractional free volume (FFV) of asphalt, which to some extent indicates that the deformation resistance of DM-modified asphalt increases with the increase of modifier admixture. The binding energy between DM modifier and base asphalt decreases with increasing modifier admixture, which to some extent indicates that the compatibility and thermal storage stability of DM-modified asphalt decreases with increasing modifier admixture. It is worth noting that 1% is still an acceptable amount at the microscopic level.

With the further spread of COVID-19 and the gradual awakening of people’s awareness of protection, a large number of masks will be produced and used. This puts tremendous pressure on the environment. The research in this paper provides a new idea for the recycling of discarded masks and recommends a suitable modifier dose to maintain the stability of the modified asphalt properties of discarded masks. The improvement method of thermal storage stability of discarded-mask-modified asphalt and its application in asphalt mixtures will be explored in future research to further promote the road use of discarded masks.

## 5. Patents

**Utility model patent:** A fast and convenient pre-treatment recycling device for waste masks; inventors: Yuanle Li, Hainian Wang, Ziye Ma, Peng Zhang, Hu Jiao, Shenglan Liu, Huimin Wang, Ponan Feng; Patent No. ZL 2021 2 2926656.4

## Figures and Tables

**Table 1:** Main indexes of asphalt.

Item	Result	Requirement
Penetration at 25 °C (0.1 mm)	74	60–80
Penetration index	−1.4	−1.5 to 1.0
Softening point (°C)	46	≥45
Ductility at 10 °C (cm)	30	≥25
Density at 15 °C (g/cm^−1^)	1.039	Measured records
Dynamic viscosity at 60 °C (Pa·s)	182	160

**Table 2:** The SARA fractions of asphalt binder models.

Group		Molecular Formula	Molecular Formula Quality (g/mol)	Quality Percentage (%)	Number of Molecules
Asphaltene	Asp-1	C42 H54 O	574.893	17.1	9
	Asp-2	C51 H62 S	707.117		6
	Asp-3	C66 H81 N	888.381		9
Saturate	Sa-1	C30 H62	422.826	10.6	12
	Sa-2	C35 H62	482.881		12
Aromatic	Ar-1	C35 H44	464.737	30.6	33
	Ar-2	C30 H46	406.698		39
Resin	Re-1	C40 H59 N	553.919	41.7	12
	Re-2	C40 H60 S	572.980		12
	Re-3	C18 H10 S2	290.398		15
	Re-4	C36 H57 N	503.859		12
	Re-5	C29 H50 O	414.718		45

**Table 3:** Density of asphalt model (298 K).

Item (g/cm^3^)	Base Asphalt	DM1	DM2	DM3	DM4
density	0.964	0.963	0.96	0.959	0.958

**Table 4:** The fractional free volume of asphalts (298 K).

Item	Base Asphalt	DM1	DM2	DM3	DM4
Occupied volume	144,143.22	145,541.47	146,794.95	148,547.39	149,565.24
Free volume	33,517.26	33,174.82	32,804.71	32,500.49	32,517.83
FFV	18.87	18.56	18.27	17.95	17.86

**Table 5:** The fractional free volume of asphalts (331 K).

Item	Base Asphalt	DM1	DM2	DM3	DM4
Occupied volume	144,147.15	145,214.31	146,586.7	148,169.41	149,491.37
Free volume	36,912.7	35,109.69	35,221.84	35,431.99	35,546.49
FFV	20.39	19.479	19.37	19.30	19.21

**Table 6:** Binding energy of DM-modified asphalt (298 K).

Dosage	Energy (kcal/mol)			
	DM-Modified Asphalt	Base Asphalt	PP	Binding Energy
1%	40,298.764	40,281.658	85.422	68.316
2%	40,496.908	40,281.658	181.537	−33.713
3%	40,608.717	40,281.658	262.520	−64.539
4%	40,789.203	40,281.658	330.748	−176.797

**Table 7:** Binding energy of DM-modified asphalt (331 K).

Dosage	Energy (kcal/mol)			
	DM-Modified Asphalt	Base Asphalt	PP	Binding Energy
1%	44,471.8077	44,341.429	141.495	11.116
2%	44v667.35295	44,341.429	248.974	−76.950
3%	44,823.86759	44,341.429	380.223	−102.216
4%	45,115.72166	44,341.429	505.819	−268.474

## Data Availability

Not applicable.
